# Plate size and food consumption: a pre-registered experimental study in a general population sample

**DOI:** 10.1186/s12966-019-0826-1

**Published:** 2019-08-28

**Authors:** Daina Kosīte, Laura M. König, Katie De-loyde, Ilse Lee, Emily Pechey, Natasha Clarke, Olivia Maynard, Richard W. Morris, Marcus R. Munafò, Theresa M. Marteau, Paul C. Fletcher, Gareth J. Hollands

**Affiliations:** 1 Behaviour and Health Research Unit, University of Cambridge, Cambridge, UK; 2 Department of Psychology, University of Konstanz, Constance, Germany; 3 UK Centre for Tobacco and Alcohol Studies, School of Psychological Science, University of Bristol, Bristol, UK; 4 MRC Integrative Epidemiology Unit (IEU), UK Centre for Tobacco and Alcohol Studies, School of Experimental Psychology, University of Bristol, Bristol, UK; 5 Department of Population Health Sciences, Bristol Medical School, University of Bristol, Bristol, UK; 6 Department of Psychiatry, University of Cambridge, Cambridge, UK

**Keywords:** Plate size, Food consumption, Choice architecture, Nudging, Physical micro-environment

## Abstract

**Background:**

There is considerable uncertainty regarding the impact of tableware size on food consumption. Most existing studies have used small and unrepresentative samples and have not followed recommended procedures for randomised controlled trials, leading to increased risk of bias. In the first pre-registered study to date, we examined the impact on consumption of using larger versus smaller plates for self-served food. We also assessed impact on the underlying meal micro-structure, such as number of servings and eating rate, which has not previously been studied.

**Methods:**

The setting was a purpose-built naturalistic eating behaviour laboratory. A general population sample of 134 adult participants (aged 18–61 years) was randomly allocated to one of two groups varying in the size of plate used for self-serving lunch: large or small. The primary outcome was amount of food energy (kcal) consumed during a meal. Additionally, we assessed impact on meal micro-structure, and examined potential modifying effects of executive function, socio-economic position, and sensitivity to perceptual cues.

**Results:**

There was no clear evidence of a difference in consumption between the two groups: Cohen’s *d* = 0.07 (95% CI [− 0.27, 0.41]), with participants in the large plate group consuming on average 19.2 (95% CI [− 76.5, 115.0]) more calories (3%) compared to the small plate group (large: *mean* (*SD*) = 644.1 (265.0) kcal, versus small: 624.9 (292.3) kcal). The difference between the groups was not modified by individual characteristics. There was no evidence of impact on meal micro-structure, with the exception of more food being left on the plate when larger plates were used.

**Conclusions:**

This study suggests that previous meta-analyses of a low-quality body of evidence may have considerably overestimated the effects of plate size on consumption. However, the possibility of a clinically significant effect – in either direction – cannot be excluded. Well-conducted trials of tableware size in real-world field settings are now needed to determine whether changing the size of tableware has potential to contribute to efforts to reduce consumption at population-level.

**Trial registration:**

The study protocol (https://osf.io/e3dfh/) and data analysis plan (https://osf.io/sh5u7/) were pre-registered on the Open Science Framework.

**Electronic supplementary material:**

The online version of this article (10.1186/s12966-019-0826-1) contains supplementary material, which is available to authorized users.

## Introduction

If people consumed less food and energy it would help to prevent weight gain and reduce the global burden of non-communicable diseases such as cardiovascular disease, cancer, and type 2 diabetes [1] which cause a large proportion of deaths worldwide [2]. Changing cues in our immediate physical environments that influence consumption could contribute to addressing this problem [3, 4]. One such intervention that has received considerable research and public attention is providing consumers with smaller tableware, such as plates or bowls, in order to reduce their intake of food. However, the likely efficacy of this action is unclear.

Recent systematic reviews and meta-analyses have reached slightly different conclusions about whether the size of the tableware – for example, plates or bowls – has an effect on the amount of food that is consumed. Robinson and colleagues [5] concluded that there was no consistent effect of larger plate size on food intake (with point estimate effects ranging from − 0.25 to + 0.96, and a pooled effect size estimate of *d* = 0.18), while a Cochrane review of the impact of portion, package and tableware size [6] identified a small to medium magnitude effect of larger tableware on consumption (*d* = 0.29). The most recent meta-analysis by Holden, Zlatevska, & Dubelaar [7] indicated a large effect of plate size on amount consumed when food was self-served (*d* = 0.70), but little effect when portion size was held constant and served on differently-sized plates (*d* = 0.03). The majority of the studies included in these systematic reviews have been assessed as being of poor quality, with the Hollands et al. [6] Cochrane review assessing all studies that manipulated tableware size as being at high or unclear risk of bias. This was due to studies not following recommended procedural guidance for conducting and reporting randomised controlled trials, for example by failing to adequately implement randomisation procedures, and conceal allocation to participants and formally register study protocols. Finally, previous studies of tableware have typically been small – with sample sizes ranging from 18 to 68 participants in studies that measured self-served food consumption – and conducted in samples that do not represent the general population, such as undergraduate students [7].

Furthermore, a substantial proportion of the studies common to these reviews have been conducted by researchers at the Cornell University Food and Brand Lab, whose work has recently been subject to scrutiny for possible scientific misconduct [8, 9]. A number of their studies have already been retracted or corrected, including a study concerning tableware size [10], suggesting the need to apply due caution to other research originating from this group. Given the reproducibility concerns highlighted across the behavioural and medical sciences in recent years, this also emphasises the need for future research to accord with principles of reproducible science, including pre-registration of protocols and statistical analysis plans, as well as open data [11].

In sum, there remains considerable uncertainty about the effects of plate size on food consumption, and high-quality pre-registered studies are needed to address this. Here we focused on consumption when food was self-served, as previous literature has suggested that this is where effects are most likely to be observed. The primary research question for this study was therefore: What is the impact on consumption of using larger (versus smaller) plates for self-serving food? In line with the weight of current review evidence, we hypothesised that using larger (versus smaller) tableware when self-serving food increases consumption of that food.

In addition, the potential mechanisms underlying any effect of plate size are not well understood. In particular, to our knowledge, no studies of the effect of plate size have examined possible impact on meal micro-structure – the pattern of behaviours that occur within an eating episode, such as number of helpings served or eating rate – that could explain any observed effects on amount consumed. In exploring potential mechanisms, the current study also examined possible modifiers of any observed intervention effect of plate size, with a secondary research question asking whether *i.* executive function, *ii.* socio-economic position (SEP), and/or *iii.* Sensitivity to perceptual cues, modify the effect of plate size on consumption. It has been hypothesised that because interventions that manipulate environmental cues do not rely on people consciously engaging and forming intentions to change their behaviour, their effects will not be moderated by executive function, specifically response inhibition. Because executive function resources are patterned by SEP, tending to be lower in lower SEP groups [12, 13], this may also mean that SEP would not moderate the effect of such interventions (although SEP could modify these associations in other ways). The purpose of examining moderation by these two variables is therefore to give a preliminary indication of the intervention’s potential to change behaviour in a way that does not exacerbate health inequalities, as an absence of moderation would suggest the intervention is similarly effective in those with varying levels of cognitive resource [4, 12]. Regarding sensitivity to perceptual cues, being more sensitive to external (relative to internal) cues could feasibly result in manipulations of environmental cues such as plate size having a greater impact, although to our knowledge no previous research has directly tested this.

## Methods

Ethics approval was obtained from the Cambridge Psychology Research Ethics Committee (PRE.2018.036). The study protocol was pre-registered on the Open Science Framework (https://osf.io/e3dfh/) before any data were collected. The data analysis plan (https://osf.io/sh5u7/) was posted prior to the completion of data collection and before any inspection of the data.

### Study design

In a two-group between-subjects design, participants were randomly allocated to self-serve lunch and eat from either a *i.* small or *ii.* large circular dinner plate. Randomisation was conducted by an external statistician not involved with data collection, using a random number generator. Participants’ allocation to experimental condition was concealed from the research team until after the participant had consented to take part in the study. The staff implementing the study procedures concerning the experimental manipulation and collection of the primary outcome were employees of the eating behaviour laboratory and were not part of the study research team, although it was not possible to blind them to the manipulation. Participants were blinded to the study conditions, and the external statistician completing the data analysis was blinded to allocation.

### Sample and setting

A general population sample varying in socio-economic position (SEP) was recruited via a research agency during the study period (August to November 2018). The sample purposefully comprised similar proportions of men and women and similar proportions of participants with lower and higher education level (no Bachelor’s degree versus Bachelor’s degree or higher). The following inclusion criteria were applied: age 18–60; sufficient English and computer literacy skills to complete the study. The exclusion criteria comprised: disliking or restricting of the test foods [e.g. food allergies, vegan]; having a diagnosed eating disorder or taking prescribed medication that could considerably influence eating behaviour; performing vigorous exercise for more than 10 h a week; active smokers. Participants were reimbursed £50 for completing the study.

Sample size was determined based on the most recent meta-analysis of tableware size and consumption [7], using G*Power software [14]. An effect size of *d* = 0.50 was assumed, which is conservative relative to the estimate from the meta-analysis (analysis of studies of self-served consumption produced an effect size of *d* = 0.70). With power = 0.80, and alpha = 0.05 for the effect on the primary outcome of larger versus smaller circular plates, a total sample of 128 participants was required. To account for participant dropout, we planned to over-recruit by 5%, meaning 134 participants were to be randomised.

The study was conducted in the Wellcome-MRC Translational Research Facility (TRF) in Cambridge, UK, a purpose-built eating behaviour facility that includes rooms designed to replicate eating environments in people’s homes (see Procedure for further details).

### Materials

#### Plates

White, circular, unmarked plates with a 46% difference in surface area were used for self-serving food: Large plate: China by Denby Dinner Plate (29 cm diameter, surface area = 660.5 cm^2^); Small plate: China by Denby Dessert/Salad Plate (23 cm diameter, surface area = 415.5 cm^2^). The plates were purposefully selected from a product range with plain, featureless designs, and that offered both a standard dinner plate and a smaller meal plate that was otherwise identical. These plate types were chosen due to public and research interest in the potential impact on consumption of reducing the size of dinner plates.

#### Food

A vegetarian cheese and tomato pasta bake (150.33 kcal per 100 g) was the default food provided. If a participant was unwilling to consume it for any reason, chicken korma curried rice (129.6 kcal per 100 g), matched in nutritional characteristics to the pasta dish, was offered as an alternative.

#### Weighing balance

An A&D GX6100 6100 g × 0.01 g laboratory balance was used to weigh the study food.

#### Video cameras

Two cameras were used to record the eating session (Silverlabel Focus Action Cam Ultra HD; Go Pro Hero 3). Participants consented to being filmed within the facility although the cameras were concealed throughout.

### Measures

#### Primary outcome: energy consumed

The amount of food consumed was measured in grams, weighing the food taken from the serving dish minus the amount of food left on the participant’s plate. This was translated into energy consumed (kcal), which is the measure used in the analysis.

#### Additional measures

*Demographic measures*: Age and gender were recorded in a study questionnaire before lunch. Weight and height were measured at the end of the study session (in order not to prime weight or diet-related concerns) to assess participants’ body mass index (BMI = kg/m^2^).

*Hunger and fullness*: We measured participants’ ratings of hunger and fullness before lunch on two separate 100 mm Visual Analogue Scales (VAS) [15] to compare whether levels were similar between the two groups.

#### Effect modifiers (assessed pre-intervention)

*Executive function – response inhibition and impulsivity*: To assess response inhibition, a stop-signal task [16] was used, in which participants are presented with an arrow within a circle that either points right or left. Participants are required to press a corresponding key for each direction unless a signal is played after the presentation of the arrow. In this case, the response should be stopped before execution. Stop-signal reaction time (SSRT), measured in milliseconds (ms) refers to the time required to stop the initiated go-process, with slower SSRT indicating poorer inhibitory control. Of the wide range of neurocognitive measures that are available, stop-signal tasks have shown relatively consistent relationships with BMI and eating behaviour including laboratory food intake [17]. Additionally, the total score of the Barratt Impulsiveness Scale (BIS-11) [18] was used to assess impulsiveness. The scale (30 items) was found to be reliable in this sample (*α* = 0.80).

*Socio-economic position (SEP)*: Three different SEP measures were collected: i) highest educational qualification, ii) household income and iii) Index of Multiple Deprivation (IMD). The latter is an official measure of the relative deprivation of geographic areas in England, [19] and was assessed for each participant by their postcode; the lower the IMD number, the more deprived the area the participant lives in.

*General sensitivity to perceptual cues – spatial orientation ability*: The Penn Line Orientation Test (PLOT) [20] computer task was used to assess participants’ ability to orient objects in space. In each trial, participants were shown a pair of lines with different orientations and were asked to rotate a moveable line to be parallel to the fixed line. There are a total of 24 trials in the test, with number of correct orientations being the measure used.

#### Meal micro-structure

While eating, participants were filmed using two concealed cameras, one of which was directly pointed towards the front of the participant’s chair. Two researchers (DK and LK) independently verified the number of helpings and number of bites from the videos, and interrater agreement was calculated (*see Results*). Based on these data, the duration of the meal (time stamp of last bite minus time stamp of first bite), average bite size (total amount of food consumed divided by number of bites; in grams), average bite duration (total duration of meal divided by number of bites; in minutes) and eating rate (number of bites divided by total duration of the meal) were calculated. Additionally, we recorded the total amount of food self-served and amount of food left on the plate.

#### Measure not included in the analysis

We assessed hedonic experience with a single item question “How did you like the food?” with an answer on a 100 mm VAS [21] anchored at “not at all” and “extremely”.

### Procedure

To conceal the true purpose of the study, at the time of recruitment, participants were informed that the study was examining the impact of time of the day on a range of mental processes, and that they had been allocated to a lunchtime session. All data were collected in individual experimental sessions that took place between approximately 12:00 to 14:45 to ensure similar hunger levels. Participants were instructed to consume one of three suggested breakfasts and fast for at least three hours before the study session. After providing written informed consent, participants initially completed a series of baseline measures in a testing room, including the computer-based cognitive tasks. Participants were then guided to a lounge room with a small dining table, a chair, a sofa, bookshelves, a television showing a standardised nature programme, and a heated food trolley with food (see Additional file 1). Participants were presented with a large serving dish containing the food, along with a plate (as determined by random allocation) and utensils. Participants were told to serve themselves and consume as much or as little food as they wished to over a given period of 30 min, eating at their own pace and serving themselves as many times as they like. During the meal, participants were allowed to have 100 ml of water, although participants who requested more water during the meal were given an additional 100 ml. Participants were on their own during the lunch and covert cameras in the room captured participants eating. Following the allocated eating time, participants were moved to another testing room where they completed the post-intervention measures (weight, height, and questions to probe the effectiveness of the cover story) and were fully debriefed about the purpose of the study.

### Data analysis

Analysis was conducted by an analyst not involved in the collection of the data and who was blinded to allocation. Descriptive statistics were calculated for baseline characteristics of participants in the two plate size conditions. SPSS 25 software was used for data analysis.

The primary outcome (energy consumed) was analysed using an independent samples t-test. Sensitivity analysis was performed after outliers for the primary outcome were removed (i.e. participants who exceeded a distance of 1.5 times the interquartile range (IQR) below the first quartile (applying to no participants) or 1.5 times the IQR above the third quartile (equating to 1250 kcal and applying to five participants). Mean differences and effect size (Cohen’s *d*) are reported alongside 95% confidence intervals (CI).

A two-way analysis of variance (ANOVA) was applied to estimate the impact of the effect modifiers (executive function, SEP and sensitivity to perceptual cues), and the interaction between plate size and each dichotomised covariate in turn. Executive function variables and sensitivity to perceptual cues were dichotomised at the median. For SEP measures, income and Index of Multiple Deprivation (IMD) were split at the median, while highest qualification was dichotomised as ‘Bachelor’s degree-level and higher’ versus lower. Interrater agreement for meal micro-structure measures was determined using intraclass correlation coefficients (ICC) in R (version 3.5.1) with package irr (version 0.84) [22]. Mean differences between groups for these measures were analysed using independent samples t-tests.

## Results

### Sample characteristics

**Fig. 1:**
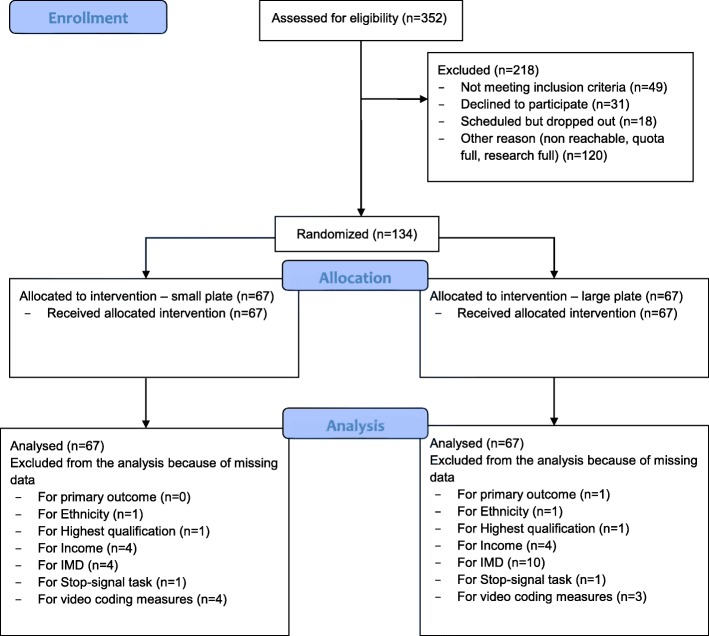
CONSORT Flow Diagram

A total of 134 participants consented to participate and were randomised. Mean age was 35.9 (*SD* = 11.9) years. As specified in the recruitment quotas, participants were near-equally split between men and women (49% men, 51% women, 1% other) and highest qualification (53% had a Bachelor’s degree or higher; 47% did not have a degree). See Table 1 for full participant characteristics, showing that baseline characteristics were well-balanced between groups. The primary outcome analysis used a sample of *n* = 133, as primary outcome data for one of the participants were missing due to an administrative error. No video data were available for six participants due to technical errors, so analyses for all meal micro-structure measures were conducted with a sample of *n* = 127, with the exception of average bite size, which used a sample of *n* = 126. Missing data points for other variables including effect modifiers are reported in Table 1. The study CONSORT flow diagram is provided in Fig. 1. Nearly all participants (128/134; 96%) followed the instruction to consume a suggested breakfast and fast for three hours before attendance.

**Table 1:** Participant characteristics

Measure	Small Plate (*n* = 67)	Large Plate (n = 67)
Gender, *n* = 134		
Men, n (%)	33 (49%)	32 (48%)
Women, n (%)	33 (49%)	35 (52%)
Other, n (%)	1 (1%)	0
Age (Mean (SD)), n = 134	35.9 (12.9)	35.9 *(*10.9)
Ethnicity, *n* = 132		
White, n (%)	57 (86%)	57 (86%)
Non-white, n (%)	9 (14%)	9 (14%)
BMI (Mean (SD)), n = 134	27.3 (4.9)	26.5 (4.7)
*Socio-economic position*		
Highest qualification^a^, n = 132		
No qualifications	3 (5%)	3 (5%)
Up to 4 GCSE’s	7 (11%)	7 (10%)
5 or more GCSE’s or 1 A-level	10 (15%)	11 (17%)
2 or more A-levels	12 (18%)	9 (14%)
Bachelor’s degree	16 (24%)	16 (24%)
Post-Graduate degree or qualification	18 (27%)	20 (30%)
Income per year before tax, *n* = 126		
Lower income		
(Up to £39,999)^a^, n (%)	29 (46%)	32 (51%)
Higher income		
(£40,000 and more), n (%)	34 (54%)	31 (49%)
IMD^b^ (Mean (SD)), *n* = 120	12.6 (7.7)	11.6 (7.2)
Hunger (Mean (SD)), n = 134	52.4 (25.8)	50.1 (23.7)
Fullness (Mean (SD)), n = 134	26.7 (24.5)	27.9 (21.8)

^a^Lower/higher income categorisation is equivalent to average household income below, and above, ~$50,000 or €45,000

^b^Index of Multiple Deprivation

### Primary outcome

There was no clear evidence of a difference in calories (kcal) consumed between the plate size groups: *t*(131) = 0.397, *p* = 0.692. Participants using large plates consumed a mean amount of 644.1 kcal (*SD* = 265.0), versus 624.9 kcal (*SD* = 292.3) for those in the small plate condition. The mean difference was 19.2 cal (95% CI [−76.5, 115.0]), equivalent to a 3% difference between groups. The effect size was very small (*d* = 0.07; 95% CI [− 0.27, 0.41]) in the hypothesised direction, with wide confidence intervals that include the possibility of a small to medium effect in either direction. Sensitivity analysis after removing outlier participants (*n* = 5) did not alter the results, with a mean difference between groups in the same direction of 40.9 cal (95% CI [− 37.7, 119.5]); *t*(126) = 1.03, *p* = 0.305; *d* = 0.18 (95% CI [− 0.17, 0.53]).

### Meal micro-structure

The only meal-microstructure measure that differed between the two groups was the amount of food left on the plate after the meal, with participants using large plates leaving 8.6 g (95% CI [1.1, 16.0]) more food. No other group differences were found for meal micro-structure measures (*t*s(*df*s ≥ 124) ≤ |1.33|, *p*s ≥ 0.186). The average time spent eating was approximately eight and a half minutes, with none of the participants still eating at the end of the allotted time. *See* Table 2 *for details*. Interrater agreement for determining the number of bites and the number of servings from the videos was excellent (ICC_bites_ = 0.997, 95% CI [0.993; 0.999]; ICC_helpings_ = 0.968, 95% CI [0.931; 0.985] [23]. This high interrater agreement suggests that internal validity is good and the data from video coding adequately reflect patterns of meal micro-structure behaviours.

**Table 2:** Primary outcome and meal micro-structure

	Small plate	Large plate				
	Mean (*SD*)	Mean (*SD*)	Mean Difference	95% CI	*p*	Cohen‘s *d*
*Primary outcome*						
Kilocalories consumed (kcal)	624.9 (292.3)	644.1 (265.0)	19.2	−76.5, 115.0	0.692	0.07
Meal micro-structure						
Meal duration (min)	8.6 (4.7)	8.7 (4.3)	0.0	−1.5, 1.6	0.953	0.01
Number of servings (n)	1.8 (0.8)	1.7 (0.7)	−0.2	- 0.4, 0.1	0.186	−0.24
Amount self-served (g)	422.6 (192.7)	443.1 (174.0)	20.5	−42.5, 83.5	0.521	0.11
Amount of food left on the plate (g)	4.9 (10.5)	13.5 (28.9)	8.6	1.1, 16.0	0.024	0.40
Number of bites (n)	41.6 (23.6)	38.3 (17.0)	−3.3	−10.5, 3.9	0.366	−0.16
Average bite size (g)	11.6 (5.0)	11.7 (4.3)	0.2	−1.5, 1.8	0.852	0.03
Average bite duration (duration/ bites), (min/n)	0.2 (0.1)	0.2 (0.1)	0.0	−0.0, 0.0	0.573	0.10
Bites per minute (bites/duration), (n/min)	5.2 (2.1)	4.8 (1.8)	−0.4	−1.1, 0.3	0.216	−0.22

### Modifiers of plate size effect

There was no evidence of an interaction between plate size and any of the measured effect modifying variables on consumption (response inhibition (*p* = 0.334); impulsivity (*p* = 0.847); highest education level (*p* = 0.564); income (*p* = 0.200); IMD (*p* = 0.399); sensitivity to perceptual cues (*p* = 0.193). Once those interactions were removed from each model, there remained no evidence of a main effect of plate size for any of the models. These analyses are reported in full in Additional file 2.

## Discussion

We found, in the most robust study to date of the effect of plate size on consumption, no clear evidence of a difference in the energy consumed between groups that self-served lunch using larger or smaller plates. The effect observed was in the hypothesised direction, but very small, with 19 kcal greater consumption from larger plates. Confidence intervals around the effect include the possibility of a small to medium effect in either direction. In addition, while more food was left on the larger plates, this concerned very small absolute differences, and there was no evidence of impact of plate size on the amount of food served or any other elements of the meal micro-structure. There was also no evidence of modification by individual characteristics, namely executive function, socio-economic position, and sensitivity to perceptual cues. While we would not predict an interaction with the first two of these, and the third was speculative in the absence of existing evidence, it would be unlikely for interactions to be detected in the absence of main effects [24].

A particular strength of the current study is that it adheres to recommended practices for open and reproducible science, including pre-registration of both the study protocol and statistical analysis plan, and an appropriate sample size calculation. Unlike most studies on this topic, it also complies with guidance for conducting randomised controlled trials, including ensuring adequate randomisation procedures, allocation concealment and blinding, as well as analysis being conducted by an external analyst unaware of group allocation. Finally, it also purposefully recruited a broadly representative, general population sample. These factors in combination mean that this study provides the most robust evidence to date to address the current uncertainty about the potential impact of altering plate size.

While conducting the study in laboratory conditions has advantages in controlling how participants are exposed to the intervention and reliably measuring their responses, giving high internal validity, there are inherent limitations conferred by this setting. In particular, external validity is compromised as the setting can never completely replicate a complex, real-world environment or eating occasion. To minimise these concerns, we used a naturalistic lounge environment within a purpose-built eating behaviour facility, which allowed an environment that would closely reflect real-world conditions. Participants were free to move around the room as they wished, were not rushed – as the time allowed was intentionally more than was needed for the meal to be consumed – and they could return to the meal as many times as they liked. A further limitation of the study was that while we were able to measure the total weight of the food consumed and observe eating behaviour throughout via video recordings, we were unable to assess all meal micro-structure characteristics over the duration of the session, such as the size of each individual serving where a participant served themselves on multiple occasions. This would have required monitoring food weight continually for which we did not have an unobtrusive method sufficiently inconspicuous to participants.

The finding of no or a very small effect on consumption suggests that previous meta-analyses of a low quality body of evidence may considerably overestimate effects [5–7]. Although the effect size we observed does fall within the 95% confidence intervals around the effect on consumption reported by the Hollands et al. [6] and Robinson et al. [5] meta-analyses, Holden et al. [7] report an estimated effect size of *d* = 0.70 for those studies that that were most similar to ours, being those that focus on self-served consumption. This represents a large effect that lies outside the confidence intervals for our study. Closer examination of effect size estimates observed in individual studies within these meta-analyses suggests that our result is consistent with most previous research. Of 12 such comparisons included in the most recent Holden meta-analysis, with point estimates of effects ranging from *d* = − 0.47 to *d* = 1.15, six comparisons report 95% confidence intervals which include the effect size seen in our study [25–27], while two found modest effects in the opposite direction [28, 29]. Four studies found large effects of increasing tableware size on consumption [25, 30–32], thus shaping the summary effect sizes in these meta-analyses. Notably, three of these latter four studies originate from the Cornell University Food and Brand Lab [30–32], meaning that due caution should be applied to their interpretation [9].

Given the varying effect size estimates accompanied by wide confidence intervals seen in single studies and meta-analyses to date, uncertainty remains around potential effects of plate size on consumption. An additional complication is that a wide variety of plate sizes have been compared in previous studies. While it is possible that effects could differ for other absolute or relative size comparisons, to our knowledge there is no clear evidence that a given magnitude of increase or reduction in plate size would lead to a commensurate effect on consumption. For example, Rolls and colleagues [26] included a comparison of a 17 cm plate to a 26 cm plate (a larger difference of 9 cm in diameter) and found no effect on intake. Based on the current study, a small effect of plate size – in either direction – cannot presently be excluded. Even an effect of this magnitude – equivalent to a 3% difference in consumption – could be meaningful in relation to population health impact, however, should it be observed to persist in real-world conditions. For example, it has been estimated that any sustained reductions in energy intake of at least 24 kcal per day – an amount equivalent to approximately 1.4% of average daily energy intake for UK adults – are likely to help prevent further weight gain in the population [33]. An effect equivalent to that observed in the current study therefore has the potential to be important and could justify further examination. This is particularly the case given there are clear opportunities for public health intervention that could capitalise on effects of plate size, such as limiting the size of plates used in some food service settings [34].

The current evidence suggests that it is not feasible to conduct a single, laboratory-based study of this manipulation that is adequately powered, because extremely large sample sizes would be required. While prior effect size estimates – based on meta-analyses derived largely from poor-quality laboratory studies – suggest achievable sample sizes, these likely substantially overestimate effects. We are therefore reliant on cumulative meta-analyses of high-quality studies that minimise risk of bias, in order to reduce the uncertainty around these effects. Crucially, however, whether this has the potential to be an effect relevant to public health efforts to reduce consumption is ultimately predicated on whether effects seen in the laboratory will be observed in real-world field settings.

The effects of tableware size on food consumption in field settings are not currently known. A small number of studies in free-living conditions have been conducted, largely by the Cornell Food and Brand Lab [31, 32], but study participants are not representative of general populations and outcome measures are typically based on observations rather than objective behavioural data. Given the extremely large, unfeasible sample sizes needed to support additional laboratory studies, this suggests that further research in this area would most appropriately consist of well-conducted trials of tableware size in real-world field settings in free-selecting food service settings. This would provide necessary information as to whether changing the size of tableware has the potential to contribute to efforts to reduce consumption at population level. The results of the current study also suggest that research on tableware size should not be considered an immediate research priority relative to other interventions that can be applied in similar food service settings. For example, reduction of portion sizes has a more robust evidence base from a range of settings, accompanied by evidence of underlying mechanisms [35–39], while reducing availability of less healthy food options is supported by preliminary evidence from real-world settings that suggests substantially larger effects [40].

Finally, we note that the use of smaller plates is widely promoted as a strategy for losing weight, including by reputable sources of healthcare advice (https://www.nhs.uk/live-well/healthy-weight/12-tips-to-help-you-lose-weight/). The current evidence is, in our view, insufficiently conclusive to either endorse or refute such advice. While the results of the current study may reduce expectations that the effect sizes previously estimated by evidence syntheses could be realised, we do not have clear, robust evidence that smaller plates are ineffective or actively harmful. Furthermore, we are not aware of relevant evidence to suggest that using an ineffective strategy reduces the likelihood of a person also using more effective approaches to reduce consumption, although this is a plausible hypothesis. This uncertainty will likely be reduced by more robust studies and the updating of high-quality systematic reviews.

## Conclusion

The most robust study to date of plate size and consumption suggests that previous meta-analyses of a low-quality body of evidence may have considerably overestimated effects. However, the possibility of a clinically significant effect – in either direction – cannot be excluded. Well-conducted trials of tableware size in real-world field settings are now needed to determine whether changing the size of tableware has potential to contribute to efforts to reduce consumption at population-level.

## Additional files

Additional file 1: Study set-up diagram. (DOCX 170 kb)

Additional file 2: Interactions and main effects of the plate size and *i)* executive function, *ii)* socio-economic status and *iii)* sensitivity to perceptual cues on the amount of calories consumed. (DOCX 15 kb)

## Data Availability

The datasets generated and analysed during the current study are available on the Open Science Framework project page https://osf.io/k74cu/.
