# Supplementary figures and images for: Plate size and food consumption: a pre-registered experimental study in a general population sample
Source: Int J Behav Nutr Phys Act. 2019 Aug 28;16:75. doi: 10.1186/s12966-019-0826-1 (PMC6714429; doi:10.1186/s12966-019-0826-1)

**Additional File 1**

**Study set-up diagram**
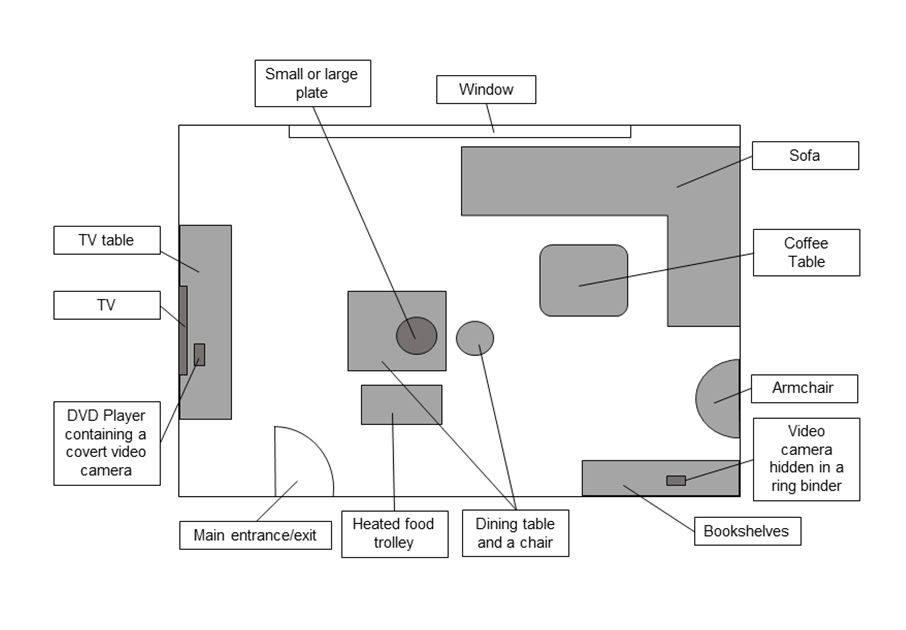

Supplement: Supplementary file 1 — Study set-up diagram. (DOCX 170 kb) [file 12966_2019_826_MOESM1_ESM.docx]
