# Supplementary material for: Plate size and food consumption: a pre-registered experimental study in a general population sample
Source: Int J Behav Nutr Phys Act. 2019 Aug 28;16:75. doi: 10.1186/s12966-019-0826-1 (PMC6714429; doi:10.1186/s12966-019-0826-1)
Supplement: Supplementary file 2 — Interactions and main effects of the plate size and i) executive function, ii) socio-economic status and iii) sensitivity to perceptual cues on the amount of calories consumed. (DOCX 15 kb) [file 12966_2019_826_MOESM2_ESM.docx]

**Additional File 2**

**Table 3. Interactions and main effects of the plate size and *i)* executive function, *ii)* socio-economic status and *iii)* sensitivity to perceptual cues on the amount of calories consumed.**

| ANOVA model | Interaction term | | | |  | Plate size main effects  (controlling for moderator, with interaction term removed) | | | | | |
| --- | --- | --- | --- | --- | --- | --- | --- | --- | --- | --- | --- |
|  | F | *p* | Interaction effect^1^ | 95% Confidence interval for interaction effect^1^ |  | F | *p* | Mean Difference (MD)^2^ | 95% Confidence Interval for MD^2^ | *d* | 95% Confidence Interval for *d* |
| Model 1: Plate size and Stop-signal task | 0.941 | 0.334 | -94.01 | -283.94, 95.93 |  | 0.048 | 0.826 | 10.65 | -85.2, 106.5 | 0.04 | -0.30, 0.38 |
| Model 2: Plate size and Impulsivity | 0.037 | 0.847 | 18.71 | -170.93, 208.35 |  | 0.113 | 0.737 | 16.2 | -79.0, 111.4 | 0.06 | -0.28, 0.40 |
| Model 3: Plate size and Highest education level | 0.335 | 0.564 | -57.04 | -250.26, 136.19 |  | 0.063 | 0.802 | 12.3 | -84.8, 109.5 | 0.04 | -0.29, 0.38 |
| Model 4: Plate size and Income | 1.662 | 0.200 | 125.16 | -65.11, 315.43 |  | 0.541 | 0.463 | 35.8 | -60.5, 132.1 | 0.13 | -0.21, 0.47 |
| Model 5: Plate size and Index of Multiple Deprivation | 0.716 | 0.399 | -86.73 | -287.67, 114.19 |  | 0.118 | 0.731 | 17.6 | -83.7, 118.9 | 0.06 | -0.28, 0.40 |
| Model 6: Plate size and Sensitivity to perceptual cues | 1.714 | 0.193 | -126.01 | -314.67, 62.65 |  | 0.186 | 0.667 | 20.8 | -74.6, 116.2 | 0.07 | -0.27, 0.41 |

^1^All interaction effects are reported in calories and are equal to the difference in the large plate size group mean calories minus the difference in the small plate size group mean calories. ^2^All mean differences are reported in calories and are equal to the large plate size group mean calories minus the small plate size group mean calories. *d* = Cohen’s *d* effect size.
